# Salivary Biomarkers in Lung Cancer

**DOI:** 10.1155/2021/6019791

**Published:** 2021-10-13

**Authors:** Hans E. Skallevold, Evan M. Vallenari, Dipak Sapkota

**Affiliations:** Department of Oral Biology, Faculty of Dentistry, University of Oslo, Oslo 0316, Norway

## Abstract

A very low percentage of lung cancer (LC) cases are discovered at an early and treatable stage of the disease, leading to an abysmally low 5-year survival rate. This underscores the immediate necessity for improved diagnostic, prognostic, and predictive biomarkers for LC. Biopsied lung tissue, blood, and plasma are common sources used for LC diagnosis and monitoring of the disease. A growing number of studies have reported saliva to be a useful biological sample for early and noninvasive detection of oral and systemic diseases. Nevertheless, salivary biomarker discovery remains underresearched. Here, we have compiled the available literature to provide an overview of the current understanding of salivary markers for LC detection and provided perspectives for future clinical significance. Valuable markers with diagnostic and prognostic potentials in LC have been discovered in saliva, including metabolic (catalase activity, triene conjugates, and Schiff bases), inflammatory (interleukin 10, C-X-C motif chemokine ligand 10), proteomic (haptoglobin, zinc-*α*-2-glycoprotein, and calprotectin), genomic (epidermal growth factor receptor), and microbial candidates (*Veillonella* and *Streptococcus*). In combination, with each other and with other established screening methods, these salivary markers could be useful for improving early detection of the disease and ultimately improve the survival odds of LC patients. The existing literature suggests that saliva is a promising biological sample for identification and validation of biomarkers in LC, but how saliva can be utilized most effectively in a clinical setting for LC management is still under investigation.

## 1. Introduction

Lung cancer (LC) is the leading cause of cancer-related deaths globally [1]. The two main subtypes of LC are non-small-cell lung cancer (NSCLC) and small-cell lung cancer (SCLC), which account for 84% and 13% of LC, respectively [2–4]. Tobacco smoke is the single greatest risk factor of LC, though other less common risk factors include asbestos, radon, second-hand smoke, alcohol, arsenic, chromium, nickel, and polycyclic aromatic hydrocarbons [5, 6]. NSCLC can be further divided into adenocarcinoma (AC), squamous cell carcinoma (SCC), and large cell carcinoma (LCC). NSCLC has a poor five-year survival rate of 25%, often related to diagnosis of the disease at a late stage with frequent distant metastasis [4, 7]. There are two subtypes of SCLC, oat cell carcinoma, and combined-SCLC. The latter subtype is defined as SCLC with components of NSCLC [5]. SCLC has an exceptionally low five-year survival rate of less than 7% [4, 8] associated with its aggressive growth and high metastatic potential [9]. Early stages of SCLC may be treated by chemotherapy and radiotherapy, while NSCLC in its early stages may be treated successfully by surgical resection [10]. Indeed, if LC is diagnosed at an early and localised stage, the 5-year survival rate increases to 59%. Unfortunately, only 17% of all LC cases are diagnosed at this stage [4]. In order to improve treatment success in terms of reduced morbidity and mortality, early diagnosis of the disease is crucial.

Both low-dose computerized tomography (LDCT) and chest radiographs have been investigated as methods of LC-screening. In a randomized clinical trial comprising at least 53,000 heavy smokers, former and active, LDCT resulted in a 20% decrease in the LC mortality rate, as compared to the chest radiographs [11]. Consequently, several medical associations recommended LC-screening using LDCT for heavy active and former smokers [12–14]. However, LDCT-screening is not completely free from limitations, and it can result in false positive and negative results and can cause a radiation hazard. The false positive results can lead to unnecessary further testing and invasive procedures, while the false negative results can delay necessary treatment [15, 16]. As a consequence of these limitations associated with LDCT, the development of complementary screening methods is highly coveted [17]. In this regard, molecular biomarkers are increasingly recognised as key knowledge not only to better understand LC biology but also to provide earlier and more precise diagnosis and to assign patients to the best targeted treatment available so that ineffective overtreatment is avoided.

Accordingly, several tumor markers such as mRNA [18, 19], microRNAs [20], cytokines [21], antioxidant enzymes [22], and fatty acids [23] spanning across several sample types such as blood/plasma [24, 25], sputum [26], and expired air [27, 28] have been investigated in LC. Although bodily fluids, such as blood, serum, urine, and sputum, have been extensively examined as liquid biopsy for diagnostic, prognostic, and predictive markers in LC, limited data exist on saliva as a potential liquid biopsy in LC [29].

**Figure 1:**
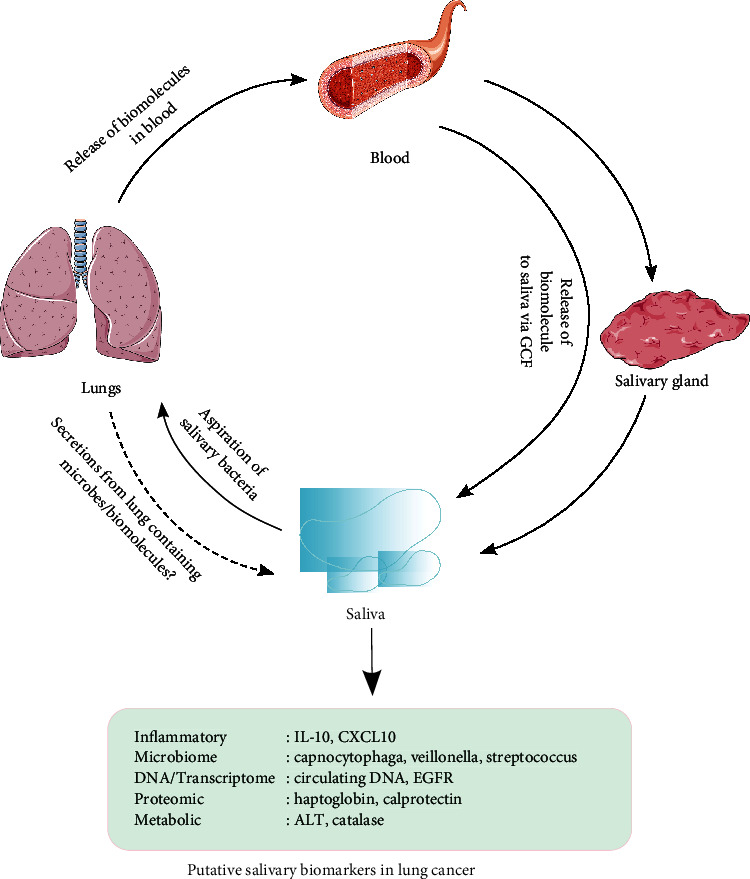
Schematic illustration showing possible pathways for enrichment of saliva for biomarkers in lung cancer [31–35].

Human saliva has been investigated as a biological fluid for diagnosis of diseases, including human malignancies. Saliva is a preferred biological sample as saliva collection is noninvasive and the procedure is quicker, cheaper, and more convenient for the patient as compared to invasive processes such as blood collection [30]. Importantly, saliva consists of a pool of biomolecules such as proteins, mRNA, miRNA, enzymes, and immunoglobulins coming from different sources, such as the salivary glands themselves [31, 32], secretions from nasal cavity and lower respiratory tract [33], gingival crevicular fluid [33, 34], and blood plasma as an ultrafiltrate [35] (Figure 1). Systemic diseases, including LC, may influence the salivary glands' function and subsequently the quantity and composition of saliva [36, 37]. In a lung cancer mouse model, a significant alteration of biomarkers in the saliva was observed. These observations suggest that tumors, even if not in close proximity, may release mediators affecting the salivary gland function and subsequently the composition of saliva [38]. In addition, saliva contains several types of bacteria, fungi, and virus species [39]. Change in the profile of these biomolecules and the microbiota in saliva in disease conditions forms the basis for the use of saliva in diagnosis and prognosis of human diseases.

The usefulness of salivary markers in both oral and systemic diseases has been investigated [40], though how markers of extraoral pathologies, like lung cancer, are found in saliva is not fully understood, and represents an important research area. This review is aimed at offering an overview of diagnostic and prognostic biomarkers in human saliva for LC (Table 1).

## 2. Methods

A literature search using the databases of PubMed and Google Scholar was performed. The search words involved the combination of the following terms from the Medical Subjects Headings (MeSH): “lung cancer,” “biomarkers,” and “saliva.” The systematic search yielded 27 articles, in the timespan from 2011 to the 31^st^ of December 2020. The inclusion criteria were as follows: (a) type of studies (human clinical studies) and (b) studies with full-text availability. The exclusion criteria were articles not related to LC and salivary biomarkers and/or articles for which full texts were not available in English. Additionally, individual articles retrieved manually from the reference list of the relevant papers were also included.

## 3. Metabolic and Inflammatory Biomarkers

Altered cellular metabolism has been identified as an emerging hallmark of cancer [41], opening an opportunity for biomarker discovery. Salivary metabolomics is a relatively new field, and accordingly, few studies have addressed the question of altered metabolic markers in saliva in cancer versus normal controls. It has, logically enough, primarily been applied to oral cancer but is increasingly expanding to more systemic diseases. The most frequently used techniques are ^1^H+NMR and mass spectrometry [42]. Bel'skaya et al. performed a comprehensive biochemical analysis of unstimulated saliva from 425 LC patients (with no prior treatment) (consisting of AC, SCC, mixed ADC+SCC, neuroendocrine, and undifferentiated LC), 168 noncancerous lung disease patients, and 550 healthy controls [43]. A major shift in salivary metabolite composition, specifically those involved in lipid peroxidation and protein metabolism, as well as metabolic enzyme activity (increased alanine aminotransferase (ALT), decreased aspartate aminotransferase (AST), and decreased AST/ALT coefficient), was observed in LC as compared to healthy controls. The change in metabolic enzyme activity was also explored previously by Bel'skaya and Kosenok [44]. Although the histological subtypes were found to have similar metabolic enzyme activities, a significant difference was observed between LC (all subtypes) and healthy controls.

Bel'skaya et al. further investigated the value of additional markers for their diagnostic utility; however, none of the investigated biochemical salivary markers could be independently used in the early diagnosis of LC. The most informative biochemical parameters were catalase activity, level of triene conjugates and Schiff bases, pH, sialic acid, alkaline phosphatase, and chloride ion concentration in saliva. This panel of seven parameters could be used to diagnose LC with 69.5% and 87.5% sensitivity and specificity, respectively. Among these parameters, Bel'skaya suggested catalase activity to be the most important parameter for LC diagnosis [43]. In addition, Fourier transform infrared spectroscopy has been examined for its utility in detecting differences in biochemical salivary parameters between LC patients and healthy subjects. Most notably in the advanced stages of LC, a significant difference was evident at infrared spectra of 1070–1240 cm^–1^ [45].

The prognostic value of salivary biochemical markers was also investigated by Bel'skaya et al. [43]. An increased concentration of lactate dehydrogenase (LDH) activity and lower imidazole (IC) concentrations were found to be significantly associated with favourable prognosis of LC. A LDH concentration of more than 1133 U/L and less than 0.311 mmol/L of IC, combined, could effectively predict a favourable outcome. Compared to patients with poor prognosis, the favourable outcomes were 1.4 (46.8% to 77.0%), 1.9 (27.1% to 47.5%), and 2.0 (18.0% to 43.3%) times more likely to survive at one, three, and five years. This was further studied in specific subtypes, and it was found that high LDH and low IC were favourable for SCC, but not for AC or neuroendocrine LC [46]. Instead, low IC levels combined with high seromucoids and uric acid were favourable for the prognosis of AC patients, and a combination of high NO, urea, and ALP was favourable for neuroendocrine tumors, as these values tend to decrease with the progression of the disease. As a predictive marker, C-reactive protein (CRP) may be of value as its concentration increases with tumor size and regional metastasis, especially in NSCLC [47].

One important caveat to using metabolic biomarkers for LC diagnosis was introduced in a systematic review of SCC of the aerodigestive tract [48]. Goh et al. found that the various classes of metabolites (branch chain amino acids, fatty acids, amino acids, carbohydrates, inorganic compounds, and lipids) showed considerable overlap in expression in LSCC, oesophageal SCC, and head and neck SCC, though predominantly between OSCC and HNSCC. In agreement with Bel'skaya et al., this further supports the need for a panel of metabolic markers, in conjunction with other proteomic and transcriptomic markers.

Inflammation is well known as both a cause and effect of tumor development. Chronic inflammation can lead to DNA damage and promote carcinogenesis. The inflammatory tumor microenvironment fosters invasion and metastatic potential of cancer cells [49, 50]. This makes inflammatory markers promising targets not only as diagnostic biomarkers but also valuable tools for determining prognosis. Several inflammation-related cytokines have been identified as significantly deregulated in NSCLC compared to healthy controls [47, 51]. Both proinflammatory and anti-inflammatory cytokines were overexpressed in the saliva of LC patients, including interleukin- (IL-) 1*β*, ILIRN, IL7, IL10, C-C motif chemokine 11 (CCL11), C-X-C motif chemokine ligand 10 (CXCL10), platelet-derived growth factor-BB (PDGF-BB), and tumor necrosis factor (TNF-*α*). Of these, the combination of IL10 and CXCL10 had the greatest diagnostic potential, with a sensitivity of 60.6% and specificity of 80.8%. The proinflammatory cytokines IL-6, IL-8, IL-18, and TNF-*α* have also been implicated in advanced LC [47].

## 4. Proteomic Biomarkers

Proteomic techniques have been predominantly used to analyse blood but have recently been adopted in salivary samples. Among such techniques are iTRAQ [52, 53] and two-dimensional gel electrophoresis (2-DE) [54, 55], which have been widely used to analyse the proteome of a number of LC subtypes. The salivary proteome has most often been profiled by two-dimensional gel electrophoresis with mass spectrometry (2DE-MS), though new techniques are being adapted to salivary proteomics as well. 2DE-MS was used in an investigation of 16 potential proteins as salivary biomarkers for early LC detection. Seventy-two subjects were enrolled in the study. The three proteins haptoglobin, zinc-2-glycoprotein, and calprotectin, combined, reached a sensitivity of 88.5% and specificity of 92.3% for diagnosis of LC [56]. Therefore, the combination of haptoglobin, zinc-*α*–2-glycoprotein and calprotectin represents a promising saliva-based diagnostic tool for LC.

Another mode of entry for biomolecules present in saliva is exosomal transport. These circulating exosomes contain lipids, proteins, and nucleic acids produced by tumor cells and can be transported in the blood as encapsulated membranes, the content of which resembles that of their parent tumor cells [57]. Sun and collaborators established a standardised method of exosome-isolation from saliva to compare their proteomic profiles. In saliva, 319 exosomal proteins were identified, along with 994 in serum, by liquid chromatography tandem mass spectrometry. Eleven exosomal proteins were discovered in saliva and plasma of LC patients that were not present in healthy subjects. This finding raises the possibility for the potential use of salivary exosomes as diagnostic biomarkers in LC [58].

## 5. Transcriptomic and Genomic Biomarkers

Several salivary transcriptomic and genomic biomarkers have received attention as molecules with diagnostic and prognostic potential. Among these are five mRNA candidates: *CCND1* (encoding for cyclin D1), *EGFR* (encoding for epidermal growth factor receptor), *FGF19* (encoding for fibroblast growth factor 19), *FRS2* (encoding for fibroblast growth factor receptor substrate 2), and *GREB1* (growth regulation by estrogen in breast cancer 1). The transcriptome signature of these genes was able to distinguish both NSCLC and SCLC from control subjects with a sensitivity of 93.75% and a specificity of 82.81% [59].

Currently, one of the most researched genetic markers for LC diagnostics is *EGFR*. *EGFR*-testing has traditionally been performed on surgically biopsied tissues. However, at the stage of biopsy taking, the LC has in most cases already progressed too far and frequent biopsies for monitoring *EGFR* mutations are impractical for these patients [60–62]. Therefore, the detection of *EGFR* by other means is highly sought after. *EGFR* is a membrane receptor frequently expressed in NSCLC that influences proliferation, angiogenesis, and chemoresistance, as well as inhibits apoptosis and promotes metastasis of NSCLC cells [63]. Identifying the presence and type of *EGFR* mutations is crucial in NSCLC patients as the common mutations, exon 19 deletion (19del) and exon point mutation 21-L858R (L858R) [64], are treatable by tyrosine kinase inhibitors such as erlotinib, gefitinib and osimertinib [65].

Electric Field-Induced Release and Measurement (EFIRM) has recently been introduced for the detection of mutations in *EGFR*. This method allows for cell-free DNA analysis using specific mutation-detecting probes, with improved sensitivity and specificity over PCR-based methods in NSCLC patients. Blood, urine, or saliva can be used as biological samples for EFIRM [66]. Two clinical studies, blinded, using EFIRM with saliva as a sample, identified the *EGFR* mutations exon 19del and L858R in NSCLC patients. The similarity between EFIRM-results and the gold standard of biopsy genotyping was high, 96-100% [67, 68]. Despite the promising results, the studies were of a small scale and need for large scale studies is evident, to explore the rate of false-positive and false-negative results [67, 68]. The method of *EGFR* detection by EFIRM fulfils many of the clinical requirements for successful and efficient detection and may become a clinical method in the future, on its own or with supplementary analysis of biopsies [67]. Another potential method of detecting *EGFR* mutations includes droplet digital PCR analysis of saliva-derived plasma cell-free DNA (plasma-cfDNA) and saliva cell-free DNA (saliva-cfDNA). No significant differences in the quantification or in concentrations of scfDNA were found between NSCLC, healthy or patients with benign lung lesions. However, the concordance rate of EGFR mutations between plasma-cfDNA and saliva-cfDNA was 83.78%[69]. Interestingly, a study by Li et al. [70] compared the concordance in detection of *EGFR* mutations of EFIRM and droplet digital PCR. The study involved thirteen patients with NSCLC, who donated plasma and saliva samples. Both *EGFR* mutations, exon 19del and L858R, were detected in both saliva and plasma samples with a sensitivity of 100%, while droplet digital PCR showed a sensitivity of 85.6% in plasma and 15.4% in saliva. The EFIRM-method was able to detect ultrashort (40-60 bp) circulating tumor-DNA fragments in saliva and plasma. This presents yet another promising and novel target for LC diagnosis in the earliest stages of the disease. In general, *EGFR* identification by EFIRM based on a simple saliva test provides high sensitivity. The method may be proven to be a great diagnostic supplement in the clinical setting.

## 6. Microbial Biomarkers

Bacterial homeostasis is important for normal bodily function, including in the oral cavity. The complex interaction involved in homeostasis of normal oral flora is considered to minimize the growth of foreign microbial invaders and opportunistic microorganisms [71]. At least 700 unique bacterial species inhabit oral cavity, though more than half are currently impossible to culture [72]. When secreted, saliva is initially sterile [73] but is quickly contaminated by bacteria shed from the surfaces of tonsils, tongue, throat, and other oral surfaces [74, 75]. The normal oral microbiome, mainly comprised of the salivary microbiome and nonshedding bacteria on supra- and subgingival dental surfaces, has largely been characterised [76, 77]. The microbial profile of saliva mirrors the composition of microbiota on oral mucosa and on dental surfaces [76, 77]. The composition of oral or salivary microbiota has been suggested to reflect oral and general health status [78].

Bacterial dysbiosis is linked to the development of a number of diseases, not only at the site of bacterial imbalance but also at distant organs. Recently, there has been a growing interest in exploring the link between salivary bacteria and the incidence and severity of respiratory infections, including COVID-19 [79, 80]. An association between periodontal disease, an inflammatory condition in the gingiva and supporting structures of teeth induced by bacteria, and several respiratory infectious conditions has been reported previously [81]. It has been suggested that oral periodontopathic bacteria can be aspirated into the lung leading to pneumonia [82, 83]. Furthermore, microbial dysbiosis at different organs and in the body fluids including that of saliva has been linked to several types of cancer, such as oral, oesophageal, colorectal and lung cancer [77, 84–88]. As an example, in colorectal cancer specimens, significantly higher levels of *Prevotella*, *Escherichia coli*, *Bacteroides fragilis*, *Streptococcus gallolyticus*, *Enterococcus faecalis*, and *Streptococcus bovis* have been detected as compared to normal colon tissues [88–90]. Similarly, in another study, significantly higher levels of *Fusobacterium nucleatum* (*F. nucleatum*) and *Clostridium difficile* were observed in patients with colorectal cancer as compared to control subjects [91]. Interestingly, enrichment of some of these bacteria such as *Prevotella* and *F. nucleatum* has been shown in oral squamous cell carcinoma specimens [87].

Several studies, using 16S rRNA sequencing technology, have reported a differential profile of salivary microbiota in LC patient as compared to the control specimens. Zhang and collaborators, using 16S rRNA sequencing technology, reported a higher richness and lower diversity of salivary microbiota in NSCLC patients as compared to that of healthy subjects [92]. The authors also reported an increase in *Veillonella* and *Streptococcus* and a simultaneous decrease of *Fusobacterium*, *Prevotella*, *Bacteroides*, and *Faecalibacterium* genera in NSCLC patients compared to the control subjects [92]. Similar findings have been reported by Yu and coworkers in NSCLC specimens [93]. In parallel to the above observation in LC specimens, Yan et al. found increased abundance of *Veillonella* and *Capnocytophaga* in saliva from LC patients (SCC and AC) as compared to that of normal controls [94]. Of note, the enrichment profile of *Veillonella* and *Capnocytophaga* in saliva was able to distinguish control subjects from lung SCC with a specificity of 86.7% and sensitivity of 84.6%, and from AC with 80% and 78.6%, respectively [94]. Interestingly, saliva from non-smoking female LC patients was reported to be enriched with *Sphingomonas* and *Blastomonas* and diminished with *Acinetobacter* and *Streptococcus* as compared to normal controls [95]. These observations, although different from other studies using saliva from LC patients, could be related to that fact that LC in non-smokers is considered to be a different disease compared to smoking related LC [54, 96–98]. Overall, these observations indicate that LC might be associated with microbiome dysbiosis in saliva and profiling of salivary microbiome might have a diagnostic value in LC.

Despite the association between microbial profile in saliva and LC as described above, the possible contribution of salivary microbiota to LC carcinogenesis is not understood. Nevertheless, salivary microbiota has been shown to influence p53 and apoptosis signalling pathways in LC tumor cells [95]. In addition, salivary microbiota has been shown to influence systemic inflammatory status in LC patients [92]. A positive correlation between *Veillonella* in saliva from NSCLC and neutrophil-lymphocyte ratio and a negative correlation between *Streptococcus* and lymphocyte-monocyte ratio have been reported. The same authors reported a decrease in folate biosynthesis and an increase in xenobiotics and amino acid metabolism in salivary metabolites from NSCLC patients [92]. Given the association between inflammation and metabolic deregulation and LC [49, 50, 92, 99, 100], the above observations indicate a possible link between dysbiotic salivary microbiota and LC carcinogenesis. However, larger and longitudinal studies are needed to clarify these suggestions.

The potential use of salivary microbiota as a diagnostic biomarker has several limitations. The microbiota is dynamic, and it continuously changes to local and systemic conditions. Moreover, its composition depends on the host's age, ethnicity, diet, oral hygiene, dental status, antibiotic use, and smoking habit [101–105]. Most of the studies so far on this topic have been conducted in Chinese population. As a result, a standardised set of microbial diagnostic markers for LC is still in its infancy.

## 7. Conclusions

Saliva as a biological sample offers several advantages. Saliva collection is a noninvasive procedure, is quicker, cheaper and more convenient for the patient as compared to invasive procedures such as blood collection. Importantly, saliva consists of a pool of biomolecules coming from different sources, such as the salivary glands themselves, secretions from nasal cavity, and lower respiratory tract. The composition of saliva is suggested to reflect local and systemic health and disease conditions. In line with this, several studies have supported a link between LC and qualitative and quantitative changes in salivary composition. Accordingly, there is a growing interest in the identification of saliva-based biomarkers in LC. Recent studies have identified a number of saliva-based protein, genomic and transcriptomic, and microbial biomarkers with diagnostic and prognostic value in LC. Among them, mutation status in *EGFR* in saliva from LC patients has emerged as potential diagnostic/prognostic marker in LC. Additionally, the salivary microbiome is a growing and fresh research area which may provide identification of microbiome-based markers in LC. However, the diagnostic and prognostic value of individual salivary markers for LC seems limited. This supports the need for identification of panel of markers, preferably combining inflammatory, genomic, transcriptomic, and microbial markers in saliva. At present, studies exploring the use of salivary diagnostic biomarkers for LC are limited to small-scale studies. Studies with larger patient groups are needed to assess salivary biomarkers' diagnostic reliability in larger and more diverse populations.

## Figures and Tables

**Table 1:** Summary of putative salivary markers in lung cancer.

	Author, year	Histological type	Sample size (LC/control)	Markers	Collection	Category	Sensitivity/specificity
Metabolic	Bel'skaya, 2020 [43]	AC, SCC, AC+SCC, NEC	425/550^∗^	Catalase activity, triene conjugates, Schiff bases, pH, sialic acid, alkaline phosphatase, chloride	Unstimulated WMS	Amino acids, biochemical	69.5%/87.5%
	Bel'skaya, 2017 [44]	AC, SCC, NEC	286/573	ALT, AST/ALT, ALP, GGT, ⍺-amylase	Unstimulated WMS	Biochemical	n/a

Inflammatory	Koizumi, 2018 [48]	NSCLC	35/35	IL-1*β*, ILIRN, IL7, IL10, CCL11, CXCL10, PDGF-BB, TNF	Unstimulated WMS	Protein	60.6%/80.8%^∗∗^

Proteomic	Xiao, 2012 [53]	Not specified	26/26	Haptoglobin, zinc-⍺-2-glycoprotein, calprotectin	Unstimulated WMS	Protein	88.5%/92.3%

Transcriptomic	Zhang, 2012 [56]	NSCLC, SCLC	32/64	*CCNI*, *EGFR*, *FGF19*, *FRS2*, *GREB1*	Unstimulated WMS	mRNA	93.75%/82.81%
	Wei, 2014 [64]	NSCLC	40/n/a	*EGFR* 19-del *EGFR* 21-L858R	Unstimulated WMS	DNA	n/a
	Pu, 2016 [65]	NSCLC other, AC, SCC	17/n/a	*EGFR* 19-del *EGFR* 21-L858R	Not specified	DNA	n/a
	Ding, 2019 [66]	NSCLC other, AC, SCC	78/26^∗∗∗^	*EGFR* 19-del *EGFR* 21-L858R	Unstimulated WMS	scfDNA^†^	n/a

Microbial	Zhang, 2019 [90]	AC, SCC	39/20	*Veillonella*, *Streptococcus*	Unstimulated WMS	16S rRNA	n/a
	Yan, 2015	AC, SCC	61/25	*Capnocytophaga*, *Veillonella*	Not specified	16S rDNA	84.6%/86.7%-78.6%/80.0%^‡^
	Yang, 2018 [92]	NSCLC	75/172	*Sphingomonas*, *Blastomonas*, *Acinetobacter*, *Streptococcus*	Unstimulated WMS	16S rRNA	n/a

^∗^Also included 168 nonmalignant lung disease cases; ^∗∗^IL10 and CXCL10 only; ^∗∗∗^also included 15 nonmalignant lung disease cases; ^†^saliva circulating free DNA; ^‡^for SCC and AC, respectively. AC: adenocarcinoma; LC: lung cancer; n/a: not applicable; NEC: neuroendocrine cancer; NSCLC: non-small-cell lung cancer; SCC: squamous cell carcinoma; SCLC: small cell lung cancer; WMS: whole mouth saliva.
